# A Portable Fluorometer for the Detection of Glyphosate

**DOI:** 10.3390/bios16040225

**Published:** 2026-04-20

**Authors:** Nathanael B. Smith, Adrian S. Rizk, Owen K. Rizk, Shahir S. Rizk

**Affiliations:** Department of Physical Sciences, Indiana University South Bend, South Bend, IN 46615, USA; nabsmith@iu.edu (N.B.S.);

**Keywords:** glyphosate, protein-based biosensors, fluorescence sensing, environmental monitoring, field-deployable diagnostics, low-cost analytical device

## Abstract

Glyphosate is the most widely used herbicide worldwide, but many current detection methods rely on lab-based chromatography, requiring costly equipment and expert users. Here, we describe a low-cost, field-deployable fluorescence biosensing platform for glyphosate detection in water and soil. An engineered variant of the *Escherichia coli* periplasmic binding protein PhnD was optimized through strategic fluorophore placement to produce a robust fluorescence signal increase upon glyphosate binding. The biosensor was integrated into a self-contained, 3D-printed device that functions as a miniature fluorometer, providing a simple yes-or-no output for non-expert users while retaining access to raw fluorescence data. The device exhibits nanomolar fluorescence sensitivity with results comparable to a benchtop fluorometer. Using this platform, glyphosate was reliably detected in buffered solutions, commercial herbicides, tap water, and soil extracts. To mitigate false positives arising from phosphate interference, we developed a dual-sensor strategy incorporating an independent phosphate biosensor and a second-generation device capable of multi-wavelength fluorescence detection. Together, these results demonstrate an affordable and versatile biosensing platform with strong potential for field-based environmental monitoring.

## 1. Introduction

Detecting environmental pollutants in soil and water is essential for safeguarding natural habitats, ensuring the health and resilience of ecosystems, and mitigating significant health threats to both human and animal populations [1]. As industrial and agricultural activities continue to expand, the pervasive presence of contaminants necessitates the development of effective monitoring strategies to assess the impact and inform remediation efforts [2]. Crucially, there is an urgent demand for affordable, portable, and user-friendly devices that can provide rapid, on-site detection of these environmental pollutants, moving beyond the limitations of traditional laboratory-bound analyses [3].

Glyphosate, a broad-spectrum herbicide, has become the world’s most widely used agricultural chemical, with widespread environmental presence raising concerns about human and ecosystem health [4,5,6]. Current analytical detection methods, including liquid chromatography-tandem mass spectrometry (LC-MS/MS) and gas chromatography-mass spectrometry (GC-MS), offer high sensitivity and specificity [7,8,9]. However, these approaches require sophisticated instrumentation, skilled personnel, and centralized laboratories, rendering them impractical for rapid field monitoring. Commercial test kits, including enzyme-linked immunosorbent assays (ELISAs) and lateral flow devices, have been developed to detect glyphosate residues in soil and water [10]. Yet, many products still rely on the end-user collecting samples and shipping them to specialized labs for analysis, with turnaround times ranging from hours to days [6]. This latency limits timely interventions in agricultural or public health settings, where immediate detection could drive rapid remediation or policy responses. Previous work has developed some field-deployable methods for the detection of glyphosate using optometric [11,12], electrometric [13,14], or fluorometric methods [15,16], as well as commercially available dip-stick type tests such as Vicam Glyphosate-V and Abraxis, each with varying levels of sensitivity and specificity.

Our laboratory has previously engineered a biosensor for glyphosate [17] based on the *Escherichia coli* periplasmic binding protein PhnD [18]. A characteristic feature of all members of the superfamily periplasmic binding proteins is a distinct conformational change upon ligand recognition [19]. Like a Venus flytrap [20], the proteins undergo hinge-bending motion from an open (ligand-free) to a closed (ligand-bound) conformation [21], making them ideal for the development of biosensors [19,22]. By attaching a fluorescent reporter to the engineered PhnD variant at position 17, we demonstrated that a large and measurable change in fluorescence can be detected upon the binding of glyphosate, providing a robust method for monitoring glyphosate concentrations [17]. While this protein-based biosensor offers a highly specific approach, its practical application has been limited by the requirement for a large and expensive benchtop fluorometer to accurately detect the fluorescence changes associated with ligand binding.

To overcome this challenge and enable widespread, accessible environmental monitoring, we designed and fabricated a novel, portable, high-sensitivity fluorometer. This compact device is specifically engineered to detect the fluorescence changes that occur when our engineered biosensor binds to glyphosate. We demonstrate that this portable device can effectively detect glyphosate in both soil and water samples, including those exposed to generic and brand-name glyphosate-containing herbicides. The device achieves a limit of detection of 3µM glyphosate, which is below the recommended maximum contaminant level for drinking water in the United States [23], ensuring its utility for public health applications. Furthermore, its user-friendly interface provides a clear, immediate “yes/no” answer, with a green light indicating a clean sample and a red light signaling contamination. The total cost of fabricating this device is under $40, utilizing readily available off-the-shelf electronic components, yet its performance is comparable to state-of-the-art fluorometers costing upwards of $40,000.

In addition to the detection of glyphosate, we developed an independent phosphate sensor based on the *E. coli* phosphate-binding protein (PBP) [24,25]. Our modified PBP, along with a modified “second-generation” multichannel fluorometer 2.0 device, provides an independent fluorescence-based sensor for phosphate in parallel to the detection of glyphosate. This dual sensor approach prevents false positives in samples contaminated with phosphate, especially in heavily fertilized soils. Given that fluorescence assays are a common and versatile method for detecting a wide array of analytes, this innovative, low-cost, and portable fluorometer platform holds immense potential for broader application in the rapid detection and monitoring of various environmental pollutants, paving the way for more proactive and widespread environmental protection.

## 2. Materials and Methods

### 2.1. Cloning and Protein Expression

All salts, buffers, and other reagents were purchased from Thermo Fisher Scientific (Waltham, MA, USA) unless otherwise indicated. The gene coding for the *E. coli* PhnD with a cysteine mutation at position 17 and a truncation of the C-terminal 9 amino acids was cloned into the pET25b+ plasmid using the restriction sites NdeI and NheI (New England Biolabs, Inc., Ipswich, MA, USA) to produce the PhnD177N∆17C construct. Cloning resulted in the addition of a C-terminal HSV-tag followed by a 6-histidine tag. The PBP164C/138A construct was cloned into pET21 using NdeI and HindIII restriction enzymes (New England Biolabs, Inc., Ipswich, MA, USA) in-frame with a C-terminal 6-histidine tag. Cloning was confirmed by DNA sequencing (Plasmidsaurus, Watterson Park, KY, USA). Protein expression was carried out by transforming *E. coli* BL21-DE3 cells (New England Biolabs, Inc., Ipswich, MA, USA) by heat shock at 42 °C for 45 s. The cells were plated on Ampicillin LB agar plates, and a single colony was used to inoculate an overnight culture of 2XYT/Ampicillin media (5 mL), which was used to inoculate a 250 mL culture of 2XYT/Ampicillin. When the cells reached an OD_600_ of 0.6–0.8, isopropyl β-D-1-thiogalactopyranoside (IPTG) was added to a final concentration of 1 mM, and the cells continued to grow for an additional 4 h at 37 °C or overnight at 18 °C. The cells were then centrifuged at 5000× *g* rpm, and the pellet was stored at −20 °C.

### 2.2. Protein Purification and Fluorescence Analysis

Protein purification was carried out as described in [17]. Briefly, pellets were suspended in ~10 mL of buffer A (50 mM TRIS-HCl, 500 mM NaCl, 10 mM Imidazole, pH 8.6), sonicated on ice, and centrifuged at 8000× *g* rpm for 30 min in the presence of lysozyme (10µM). Supernatants were loaded on a Ni-NTA agarose resin column and purified on an AKTA start using a 10 mM to 300 mM imidazole gradient. Fractions containing the protein were identified by SDS-gel electrophoresis. Purified proteins were exchanged into working buffer (20 mM MOPS, 100 mM NaCl, pH 6.9) using Econo-Pac 10 DG size-exclusion columns (Bio-Rad, Hercules, CA, USA). Fluorescence labeling was carried out by the addition of a 5–10-fold molar excess of the thiol-reactive (maleimide or iodoacetamide) fluorophore to each protein in the presence of 1 mM tris(2-carboxyethyl)phosphine (TCEP). Samples were incubated at room temperature overnight, tumbling end-to-end. Labeled proteins were isolated from excess fluorophore using Econo-Pac 10 DG size-exclusion columns. Fluorescence measurements were performed on a Jasco FP-8500 spectrofluorometer (Jasco, Inc., Easton, MD, USA). For AF488-labeled proteins, excitation was set to 480 nm, and emission spectra were collected from 500 to 580 nm. Coumarin-conjugates were excited at 419 nm, and emission spectra were recorded between 430 and 520 nm. Ligand titrations were carried out by incrementally increasing the ligand concentration and monitoring corresponding changes in fluorescence intensity. Dissociation constant (*K_D_*) values were determined by fitting the titration data from 3 experiments to a single-site binding isotherm using Kaleidagraph software (version 3.6). The equation relates the observed fluorescence (*F*) at a given ligand concentration [*L*] to the fluorescence in the absence of ligand (*F_o_*) and the fluorescence at saturating ligand concentrations (*F_max_*).

$$F=F_{o}+\frac{F_{max}-F_{o}}{1+\frac{K_{D}}{\left[L\right]}}$$

### 2.3. Design and Fabrication of Portable Fluorometer

Design of all mechanical components was carried out in Fusion 360. The central structural component was a combined cuvette-and-LED holder, which positioned the cuvette in the optical path and fixed the excitation LEDs at a defined 90° angle so that emitted light from the non-illuminated cuvette face could be directed toward two lenses placed at a set distance for focusing onto the sensor. Optical focusing was achieved using two plano-convex lenses (5 mm thick and 12 mm in diameter) mounted in printed lens holders. The electronics were distributed across two standard half-sized solderless breadboards (55 mm × 83 mm), which supported the Nano-format 8-bit ATmega328P microcontroller (Arduino, Monza, Italy), LED circuitry, a 4-pin tactile pushbutton, and the TSL2591 photodetector (Adafruit Industries, Brooklyn, NY, USA). Illumination and indicator signaling were provided by standard 5 mm diffused through-hole LEDs. All structural components were fabricated on a Snapmaker Artisan 3-in-1 3D Printer (Snapmaker, Shenzhen, China) using black PLA filament, printed at 200 °F. The printer provides a ±0.1 mm dimensional tolerance, and the assembled device was secured using standard machine screws sized for 3 mm mounting holes. CAD files and control programs are provided in the Supplementary Data.

### 2.4. Testing Water and Soil Samples

Glyphosate samples were prepared from a 50 mM stock in working buffer. Serial dilutions (1:10) were prepared for carrying out titrations. Commercial glyphosate samples were diluted as recommended by the manufacturer (1:50 dilutions) either in polished water or in tap water. For testing in the portable device, a 3 mL sample of 300 nM PhnD177N∆17C-AF488 in working buffer was placed in a clear disposable cuvette inside the cuvette holder of the device. A baseline fluorescence was collected by illuminating the blue LED and collecting the Lux values for 10 s. Average and standard deviation were collected and stored in the processor as the blank fluorescence. Then 50 µL of a suspected sample was added to the cuvette, fluorescence (Lux) was collected for another 10 s, and stored in the processor as “sample fluorescence”. The ratio of sample-to-blank fluorescence was calculated by the processor, and a red light was illuminated if the ratio was between 1.1 and 3.2, indicating contamination. If the ratio was below 1.1, then a green light illuminated to indicate a glyphosate-free sample. Ratios above the set range triggered both red and green lights to illuminate to indicate a faulty test.

For soil testing, 10 g of organic soil were sprayed with one of the following samples: tap water, diluted name-brand herbicide (Round-Up Concentrate Plus 18% glyphosate), or diluted generic herbicide (Compare N Save Concentrate 41% glyphosate). Samples were incubated overnight, then ~1 g of soil was mixed with 1 mL of working buffer and filtered through a 0.22 µm syringe filter, then one drop of the filtrate (~35 µL) was added to a cuvette containing 300 nM PhnD177N∆17C-AF488 in working buffer. Fluorescence before and after the addition of the sample extracted from the soil samples was used to determine the presence of glyphosate.

### 2.5. The 2.0 Model and Dual Sensor

The 2.0 model incorporated several major improvements including an expanded number of LED mounting positions within the cuvette/LED holder, integration of a standard *I*^2^*C* 16 × 2 LCD display, added space for a 9 V or higher-capacity battery, inclusion of a two-button membrane keypad, replacement of the original detector with the AS7341 multispectral (10-channel) sensor, and a tight-fit fixed lens casing for improved optical stability. CAD files and control programs are provided in the Supplementary Data.

A blue LED (462 nm) was used to excite the AF488-conjugated PhnD; emission was collected in the green (515 nm) channel, and a UV LED (396 nm) was used to excite the Coumarin-conjugated PBP; emission was collected in the blue (480 nm) channel. Baselines were established by collecting light at both channels sequentially for 10 s each before the addition of the sample, then collected again for 10 s each after the addition of the sample. The ratio of signal before and after the addition of the sample in each channel was calculated by the Arduino processor and reported on the LCD screen.

## 3. Results

### 3.1. Optimization of the Fluorescent Properties of an Engineered Glyphosate Binding Protein

**Figure 1:**
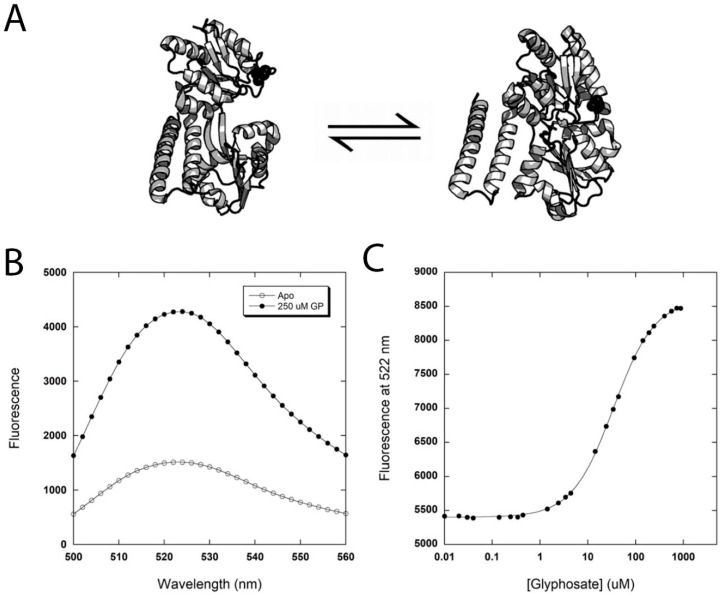
(**A**) The conformational change associated with ligand binding equilibrium. When PhnD binds to its ligand, a hinge-bending conformational change takes place, and the protein goes from an open (left) to a closed (left) form. A cysteine at position 17 (shown as black spheres) is used to attach fluorescent groups. The binding of the ligand triggers changes in fluorescence. (**B**) The biosensor, PhnD177N∆17C-AF488, shows a large increase in fluorescence (~300%) when glyphosate is added. The plot shows the fluorescence spectra in the apo (unbound) form (open circles) and in the presence of 250 µM glyphosate (closed circles). (**C**) Titration of the biosensor with glyphosate. Monitoring the change in fluorescence as a function of glyphosate showed a dissociation constant (*K_D_*) of 34 µM.

Previous work in our lab showed that the *E. coli* bacterial periplasmic binding protein PhnD can be reengineered to bind to glyphosate with high affinity and specificity [17]. Mutation of glutamic acid 177 to asparagine, as well as truncation of the last 6 amino acid residues, resulted in a 150-fold increase in the affinity for glyphosate. Attachment of a thiol-reactive fluorescent group was accomplished by the introduction of a unique cysteine at position 125. Upon ligand binding, the protein undergoes a large conformational change (Figure 1A), which in turn triggers a change in fluorescence. In particular, when thiol-reactive acrylodan or coumarin was attached at position 125, large decreases in fluorescence were observed upon binding of the protein to glyphosate. We sought to explore different positions for fluorophore attachment that would produce an increase in fluorescence when the ligand is bound. We first moved the cysteine mutation to position 17 based on previous studies [26] to produce a construct we call PhnD177N∆17C. The protein also contained a C-terminal HSV tag followed by a 6-histidine tag. The purified PhnD177N∆17C was conjugated to five different fluorophores, and changes in fluorescence upon addition of glyphosate were determined (Table 1). The PhnD177N∆17C conjugated with AlexaFluor488 (AF488) showed the largest change in fluorescence (~ three-fold increase) when saturating concentrations of glyphosate were added (Figure 1B). Monitoring the change in fluorescence of the PhnD177N∆17C-AF488 (the biosensor) as a function of glyphosate concentration was used to determine a dissociation constant (*K_D_*) of 34 µM (Figure 1C).

### 3.2. Device Design

Our aim was to develop a self-contained, easy-to-use device for an untrained, non-scientist, end-user with a yes-or-no result from a push of a button, while retaining sufficient information for an expert user to examine the raw data. Serving as a miniature fluorometer, the device must provide an excitation source with the appropriate wavelength to excite the conjugated fluorophore, have a light detector that collects the emitted light with sufficient sensitivity, and contain the capability to process the difference in fluorescence before and after a sample is added to the protein. There have been examples of low-cost fluorometer-like devices in the literature. For example, the Fluorino [27] uses an Arduino processor, an LED as a light source, and a low-cost photoresistor as the light detector in a benchtop setup. There are other examples of more portable fluorescence measurement devices [28,29,30]. Each of the examples offers certain advantages and has some limitations. None, to our knowledge, have been used for the detection of glyphosate.

**Figure 2:**
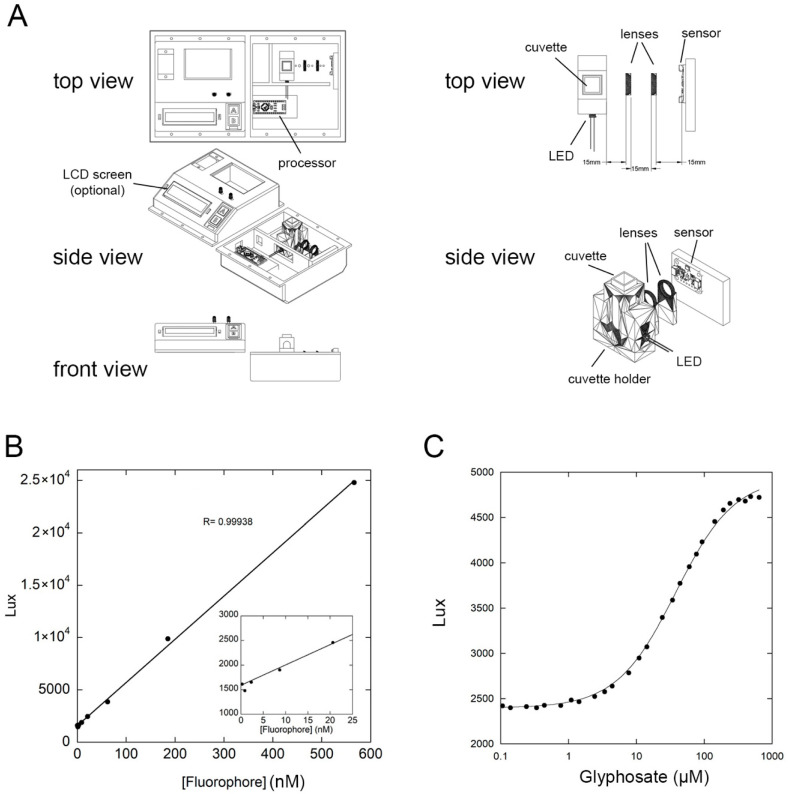
(**A**) Schematic of the device. Left: top, side, and front views showing the various components of the device. Right: top and side views of the cuvette holder, lenses, LED light source, and light detector. (**B**) Linear range of detection of the fluorophore AF488 by the device. Inset: a zoomed-in view of the low end of the plot showing the detection limit of detection down to single-digit nanomolar concentrations. (**C**) Titration of the PhnD177N∆-AF488 with increasing concentrations of glyphosate on the device by monitoring an increase in Lux as a function of glyphosate concentrations. The titration curve and the *K_D_* for glyphosate were nearly identical to those obtained by the state-of-the-art fluorometer in Figure 1C.

Here, we elected to build a self-contained 3-D printed chamber to house all of the components of the device and to block ambient light from interfering with the measurements (Figure 2A). We used a blue LED with the peak emission at 462 nm (Supplementary Data). For the detector, we used the TSL2591 photo diode sensor, a highly sensitive light detector with a wide dynamic range. We designed a 3-D printed chamber to house a four-sided clear cuvette, as well as the blue LED at a 90° angle relative to the light detector. The device also contains a 3D printed housing for a small lens between the sample and the detector to focus the emitted light. Processing is carried out by an Arduino Nano using a custom-made program that orchestrates data collection, processing, and the output to the end user (technical drawings for the 3D printed components, wiring diagram, and program are attached as Supplementary Data). The design also includes a slot for attaching an optional LCD screen (LCD 1602 module). If the screen is not used, a 3D printed cover can be used in its place to block ambient light.

To validate the performance of the device, we determined the limit of detection by testing a range of concentrations of the AlexaFluor-488 (AF488) fluorophores. We determined that the device has a linear range that spans roughly three orders of magnitude up to ~500 nM fluorophore (Figure 2B). In addition, the device can reliably detect concentrations in the single-digit nanomolar range (Figure 2B, inset). With the ability of the device to detect fluorescence changes, we carried out glyphosate titration by the addition of increasing amounts of the ligand to 300nM of the biosensor. The results from our device (Figure 2C) are nearly identical to those obtained on the state-of-the-art fluorometer with the same change in fluorescence (Figure 1C), and a similar dissociation constant (37 µM compared to 34 µM).

### 3.3. Detection of Glyphosate in Soil and Water Samples

To produce a quick yes-or-no test for glyphosate, we modified the program for the Arduino processor to collect a first baseline (blank) fluorescence, then another baseline (sample) fluorescence after the sample has been added, and then compare the two values. At the onset of the test, a disposable cuvette containing 200 to 300 nM PhnD177N∆17C-AF488 in 3 mL of working buffer (50 mM MOPS, 150 mM NaCl, and pH 6.9) is placed into the cuvette chamber within the device. Then the lid is placed over the top of the device to block ambient light. A first button-push triggers the blue LED to excite the biosensor, and emission data received by the light detector is collected every 200 msec over the period of 10 s. This establishes a “blank” baseline for fluorescence, with an average and standard deviation calculated by the processor. Then the sample suspected of containing glyphosate is added to the cuvette (typically 50 µL) and mixed. A second button push triggers LED excitation of the biosensor and another 10 s period of data collection and calculation of the average and standard deviation. The processor compares the average of the two readings (before and after sample addition) by dividing the baseline after addition of the sample by the average of the “blank”. If a ratio between a set range (1.1–3.5) is obtained showing an increase in fluorescence upon addition of the sample, then the sample is set to be contaminated with glyphosate. In that case, a red light on the outside of the device is turned on. If the ratio of the two averages is below 1.1, then the sample is said to be clear of glyphosate contamination, and an external green light turns on. Ratio greater than the set range triggers both green and red lights to turn on, indicating a faulty test.

### 3.4. Testing Samples Containing Glyphosate

**Figure 3:**
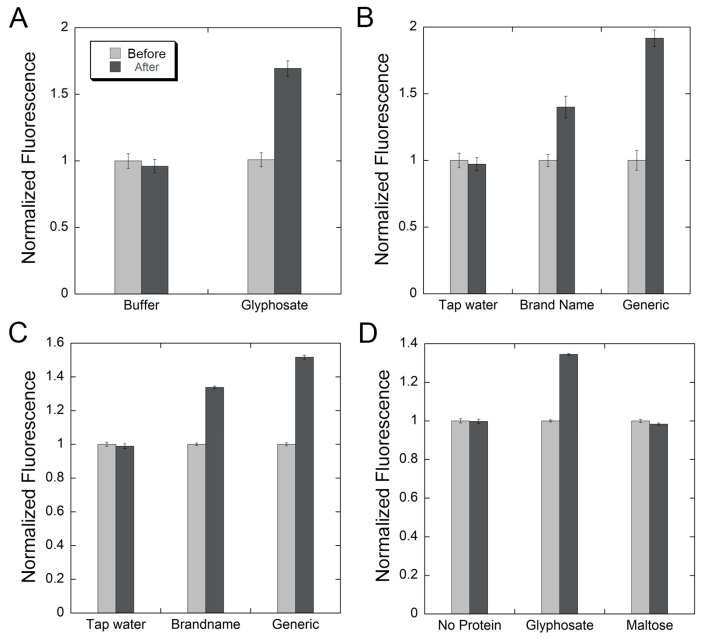
Detection of glyphosate in water and soil samples using the device. In all experiments, light output (fluorescence) of the biosensor was collected before (light gray bars) and after (dark gray bars) addition of the sample. (**A**) Samples containing glyphosate showed a large increase in fluorescence (solid lines), whereas samples with no glyphosate (buffer) showed no increase in fluorescence (dotted lines). (**B**) Tap water shows no increase in signal, whereas the brand-name herbicide (RoundUp concentrate) or the generic herbicide (Compare N Save) shows a large increase in light signal, indicating the presence of glyphosate. Both the generic and the brand-name herbicides were diluted in tap water per the manufacturer’s instructions. (**C**) Organic topsoil was treated with either tap water, the generic herbicide, or the brand-name herbicide. The samples were incubated overnight, then about 1g of the soil was removed from each treatment, mixed with 1 mL working buffer, and filtered to remove debris. Tap water soil extracts show no signal change; extracts from soil samples treated with the generic herbicide or the brand-name herbicide show a large change in signal, indicating the presence of glyphosate. (**D**) A test was performed in the absence of the protein, where glyphosate was added (no protein), resulting in no change in light output. To show that the protein is specific to glyphosate detection, a test was performed in the presence of the protein biosensor where maltose (an unrelated ligand) was added, resulting in no change in light output. A test for the detection of glyphosate was used as the positive control (glyphosate), showing an increase in light output when glyphosate was added.

To confirm that the device is capable of detecting glyphosate, we ran several tests by adding either a working buffer containing glyphosate or a working buffer alone to the biosensor protein within the device. In each test, 50 µL of the sample with or without glyphosate was added to 3 mL of the biosensor at a concentration of 300 nM. The final concentration of glyphosate in the positive samples was 170 µM. Our results show that each of the samples containing glyphosate showed a large increase in fluorescence compared to the blank baseline, whereas samples with glyphosate showed no difference (Figure 3A). In the case of samples containing glyphosate, the red LED illuminated after each test, whereas in the samples containing no glyphosate, the green LED illuminated.

In order to show that the device can function in a real-life setting, we tested its ability to detect glyphosate in commercially available herbicides. Often, these products contain other ingredients, which could potentially interfere with the sensing process or affect protein function. We tested two products, the brand-name weed killer (RoundUp Concentrate Plus, 18% glyphosate isopropyl amine salt, Bayer AG, Leverkusen, Germany) and the generic weed killer (Compare N Save Concentrate, 41% glyphosate, Ragan & Massey LLC, Ponchatoula, LA, USA). Each product was diluted in tap water, according to the manufacturer’s instructions, and 50 µL of each of the dilutions was used for testing, as was carried out for the previous glyphosate tests. We also tested tap water as a control. The results show that weed killers produce positive tests, significantly increasing fluorescence relative to the blank baseline. Both the brand-name weed killer and the generic brand showed large increases in fluorescence output (Figure 3B). No change was observed for tap water.

Because of the widespread use of glyphosate on soils in residential, commercial, and farm settings, it is important that the device is able to test soil samples for the presence of glyphosate [31]. Soil testing presents a different challenge, as debris can cause turbidity within the test, which could interfere with the passage of the light through the device or cause light scattering, ultimately obscuring the results [32]. To overcome this challenge, we devised an extract-and-filter approach. First, 10 g of organic soil were sprayed with 10 mL of nano pure water (polished water), tap water, diluted name-brand herbicide, or diluted generic herbicide. The soil samples were left overnight, then approximately 1 g of each soil sample was collected and mixed with 1 mL of working buffer inside a 10 mL syringe. The mixture was then pushed through a 0.22 µm syringe filter. One drop from each sample (~35 µL) was added to a cuvette containing 300 nM of the biosensor in 3 mL of working buffer within the device. After collecting a baseline blank and sample addition, the results show that both nano pure and tap water exhibit no change in fluorescence, whereas the samples isolated from soils treated with the brand-name or the generic herbicide showed significant increases in fluorescence, registering positive tests (Figure 3C).

To confirm that the detected changes in fluorescence are indeed due to the interaction between the biosensor and glyphosate in the suspected samples, we ran several controls. First, we conducted a test by adding a sample containing glyphosate in the absence of the protein biosensor. No change in fluorescence was observed, indicating that glyphosate by itself does not change the light output and that the protein is required for detection (Figure 3D). We also tested the specificity of the biosensor by adding different ligands to the protein. No changes in fluorescence were observed when sugar maltose was added. Taken together, these results show that the protein binds specifically to glyphosate and that this binding event triggers a detectable change in fluorescence.

### 3.5. A Dual Sensor for Phosphate and Glyphosate

While the protein biosensor binds with sufficient affinity to glyphosate, it also binds to a number of other molecules with a similar structure [17,18]. This can potentially result in a false positive test if the biosensor binds to one of the other ligands it recognizes in the absence of glyphosate. Of the ligands that can interfere with the biosensor, the most notable molecule is inorganic phosphate. While typical phosphate concentrations in water or soil are usually too low to interfere with the biosensor, phosphate concentrations may be high in heavily fertilized soils [33,34].

**Figure 4:**
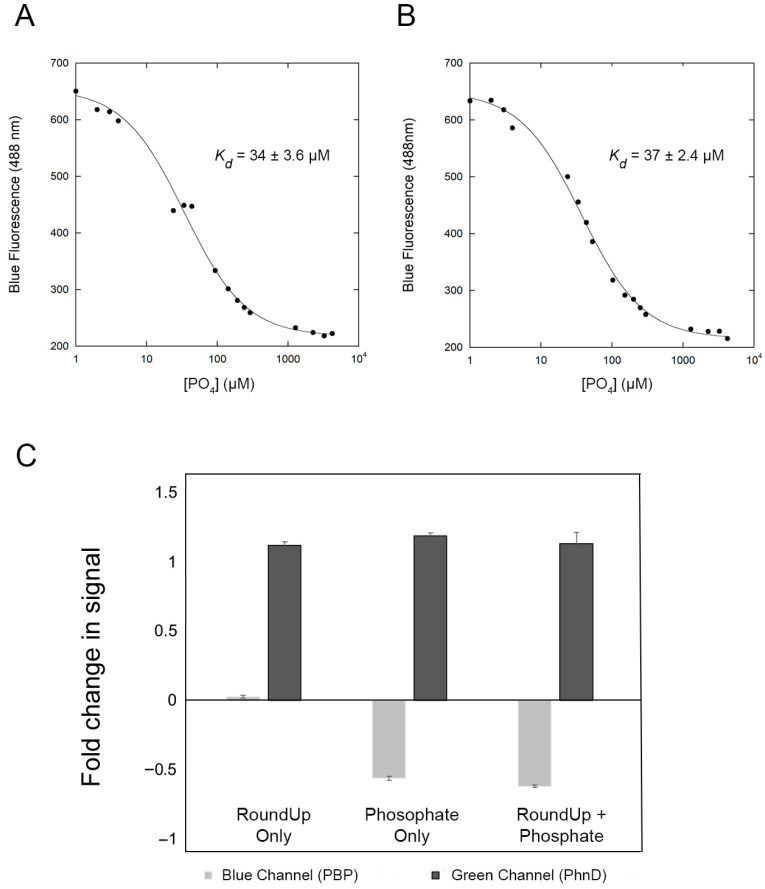
Titration of PBP164C/138A-Courmarin with phosphate in the absence of glyphosate (**A**) or in the presence of 1 mM glyphosate (**B**), showing no significant change in the *K_D_* value. (**C**) Dual sensor on the 2.0 device. Samples containing only RoundUp trigger the green channel with an increase in the fluorescence from the PhnD177N∆-17C-AF488 conjugate, but have no effect on the PBP164C/138A-Coumarin (blue channel). Samples containing phosphate only or a mixture of phosphate and RoundUp trigger both green (increase) and blue (decrease) channels.

To circumvent false positive tests due to phosphate contamination in samples, we set out to develop an independent phosphate biosensor. We used the *E. coli* phosphate-binding protein (PBP) to develop a biosensor for phosphate. Previous work shows that attaching the fluorophore Coumarin at position 164 results in a large change in fluorescence when the protein binds to phosphate [19]. However, PBP exhibits a very high affinity for phosphate [19,25], too high for field application. Therefore, we engineered a PBP variant (PBP164C/138A) that preserves the same large change in fluorescence and can detect phosphate in the micromolar range (Figure 4A). Our results show that PBP164C/138 has a dissociation constant (*K_D_*) of 34 ± 3.6µM for phosphate. Importantly, phosphate-binding is not affected by the presence of glyphosate (1mM) with a similar *K_D_* (37 ± 2.4µM) (Figure 4B).

To develop a dual sensor with the ability to independently detect phosphate and glyphosate, we sought to upgrade our initial design to a second-generation design, which we called the 2.0 device to enable simultaneous detection of fluorescence at multiple wavelengths. First, we included additional slots for up to three different LED light sources for excitation of the sample near the sample holding chamber. Second, we used the AS7341 multichannel light sensor, which can detect up to 10 independent light channels (415 nm, 445 nm, 480 nm, 515 nm, 555 nm, 590 nm, 630 nm, 680 nm, IR, and total light). Third, in place of the red/green light output, we added an LCD screen for better interaction with the user and to provide more specific written output. The screen can provide detailed instructions on sample handling and testing, show raw data as they are collected by the device, or provide final results of the test (contaminated or safe). We also redesigned the 3D printed case to include a 9-volt battery as the power source and adjusted the code accordingly to allow for the Arduino Nano processor to manage the desired tasks.

Combining the 2.0 device with the dual glyphosate/phosphate biosensor proteins, we were able to determine if a sample tests positive due to contamination with phosphate. First, a 3 mL solution containing both PhnD177N∆ labeled with AF488 and PBP164C/138A labeled with Coumarin was placed in a cuvette inside the device. Second, a baseline is collected for each protein. A UV LED (372 nm) is used to excite the phosphate biosensor, and emission is collected in the 488 nm (blue) channel. Then a blue LED (462 nm) is used to excite the glyphosate biosensor, and emission is collected in the 515 nm (green) channel. A suspected sample (50 µL) is added to the cuvette, and the measurements are repeated. Finally, the Arduino processor compares the data from both channels before and after the addition of the sample.

Unlike the glyphosate sensor, which exhibits an increase in fluorescence upon ligand binding, the phosphate biosensor shows a decrease in fluorescence when phosphate binds. When only glyphosate is found in the sample, an increase in the green channel is observed with no significant change in the blue channel (Figure 4C). But since phosphate binds to both biosensors, a sample containing only phosphate or both phosphate and glyphosate triggers changes in both channels with an increase in the green channel and a decrease in the blue channel (Figure 4C). Notably, a sample containing only phosphate with no glyphosate also triggers changes in both channels, and therefore, is indistinguishable from samples containing both phosphate and glyphosate. While this strategy does not distinguish between the presence of only phosphate or a mixture of phosphate and glyphosate, it has a clear advantage over the single sensor strategy. The dual sensor can identify if there is phosphate contamination and report a possible false positive requiring further analysis.

### 3.6. Stability and Shelf-Life of the Glyphosate Sensor

**Figure 5:**
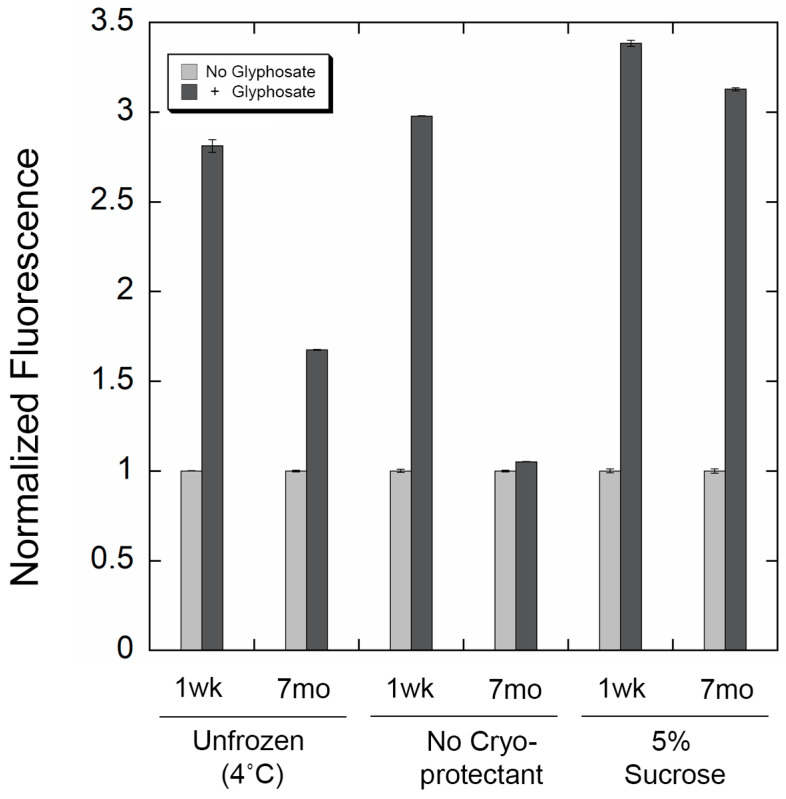
Stability of the protein biosensor under different conditions. The bars show the normalized fluorescence signal before (light gray) or after the addition of glyphosate (dark gray). We compared the signal change of 1-week-old protein (1 wk) and 7-month-old protein (7 mo) when stored at 4 °C, lyophilized with no cryoprotectant, or lyophilized in the presence of 5% sucrose. Signal change upon addition of glyphosate decreases over time with unfrozen protein or the lyophilized sample with no cryoprotectant. In contrast, the biosensor appears to maintain its signal change for at least 7 months of storage at room temperature when lyophilized in the presence of sucrose.

As is the case with most biological molecules, proteins can denature over time, affecting their activity. We observed that storing the purified, labeled PhnD biosensor at 4 °C resulted in a decrease in the overall change in fluorescence in response to glyphosate. In order for the biosensor described here to withstand field conditions and long-term storage, we sought to find conditions to stabilize the protein. Lyophilization is a widely used strategy to enhance protein shelf life by stabilizing biomolecules in the solid state [35], and the inclusion of disaccharides such as sucrose as cryoprotectants can be critical for preserving native structure and preventing aggregation during drying and long-term storage [36]. We compared unfrozen protein stability with lyophilized protein in the absence of cryoprotectant and in the presence of 5% sucrose by measuring the maximum fluorescence change in response to glyphosate (Figure 5). As expected, unfrozen protein (stored at 4 °C) showed a large decrease in fluorescence response after 7 months. The lyophilized protein in the absence of cryoprotectant showed the largest decrease in signal response. On the other hand, protein lyophilized in the presence of 5% sucrose showed nearly no change in its overall fluorescence in response to glyphosate after 7 months of storage at room temperature. This demonstrates that our biosensors can withstand at least several months of storage in lyophilized form and exhibit near full activity upon reconstitution.

## 4. Discussion

In this work, we present an integrated protein-based biosensing platform for the detection of glyphosate that combines engineered molecular recognition with a low-cost, portable fluorescence detection device. The results demonstrate that both the biosensor protein and the accompanying hardware can be optimized to achieve sensitive, user-friendly detection of a widely used environmental pollutant in water or soil samples. A central finding of this study is the successful optimization of the fluorescent response of an engineered glyphosate-binding protein. Previous iterations of PhnD-based biosensors relied on fluorophore attachment sites that produced decreases in fluorescence upon ligand binding, which can be challenging to interpret in field settings. By relocating the fluorophore attachment site to position 17 and screening multiple fluorophores, we identified a configuration (PhnD177N∆17C-AF488) that produces a robust increase in fluorescence upon glyphosate binding. This signal-on response, with a large fluorescence increase at saturation and a dissociation constant in the low-micromolar range, is particularly advantageous for practical sensing applications, as it improves signal-to-noise ratios and reduces ambiguity in interpretation. As with all periplasmic binding protein-based biosensors, the detection limit of the ligand is about 1/10 of the *K_D_* value [19,22], which, for the sensor described here, is around 3 µM (0.5 ppm), just below the allowed concentration of glyphosate in drinking water in the United States [23].

Equally important is the demonstration that this biosensor can be effectively integrated into a compact, low-cost, and field-deployable detection device. The custom-built fluorometer described here achieves sensitivity and dynamic range comparable to a state-of-the-art benchtop fluorometer, while relying on inexpensive components, simple optics, and open-source electronics. The ability of the device to detect nanomolar concentrations of fluorophore and to reproduce glyphosate binding curves obtained with state-of-the-art laboratory instrumentation underscores the feasibility of translating protein-based biosensors into practical tools for environmental monitoring.

A key strength of this platform is its emphasis on usability by non-expert end users. The development of a simple yes-or-no output, triggered by a two-button workflow and reported through intuitive LED indicators, addresses a major barrier in the deployment of biosensors outside the laboratory. At the same time, retention of raw fluorescence data and optional LCD readouts ensures that expert users can access quantitative information when needed. This dual-use design highlights how biosensing technologies can be made accessible without sacrificing analytical rigor.

The successful detection of glyphosate in commercial herbicide formulations in tap water and soil extracts shows that the biosensor remains functional in complex matrices that contain potential interferents. We also show that lyophilized protein remains active for at least 7 months of storage at room temperature. Therefore, the freeze-dried protein can be provided in a kit where the end-user can reconstitute the protein with a provided buffer solution, and use the device to measure the fluorescence before and after addition of the suspected sample. Importantly, soil testing, which is often a major challenge for optical sensing due to turbidity and scattering, was enabled through a simple filtration protocol that preserved sensor performance. In a commercial kit form, the end-user would be provided with a syringe filter that can be used to prepare a soil sample for measurement. These results suggest that the platform could be deployed for on-site screening in agricultural, residential, or environmental contexts where access to centralized laboratory testing is limited. While the change in fluorescence is reflective of the concentration of glyphosate within a range (roughly 0.1 × *K_D_* to 10 × *K_D_*), the system described here would mainly be used as a field-deployable, easy-to-use, yes-or-no test for the presence of glyphosate to quickly identify potentially contaminated samples that can then be tested with state-of-the-art gold standard methods.

Specificity is a critical consideration in pollutant detection, and our results highlight both a limitation and a solution. While the engineered PhnD variant binds glyphosate with high affinity, its ability to bind structurally related molecules, such as inorganic phosphate, has been documented previously [17,18] and raises the possibility of false positives, particularly in heavily fertilized soils. To address this challenge, we developed a complementary phosphate biosensor based on a modified phosphate-binding protein with tuned affinity in the micromolar range. By integrating both biosensors into a second-generation 2.0 device capable of multichannel fluorescence detection, we established a dual-sensor strategy that can flag potential phosphate interference. Although this approach does not fully deconvolute mixed glyphosate–phosphate samples, it represents a significant improvement over single-sensor designs by identifying cases where additional confirmatory analysis is warranted.

Taken together, these findings have important implications for the broader field of environmental pollutant detection. Conventional methods for glyphosate analysis, such as chromatography and mass spectrometry, are highly sensitive but require expensive instrumentation, trained personnel, and centralized laboratories. In contrast, the platform described here offers a complementary approach focused on rapid screening, affordability, and deployment at the point of need. Such tools are especially valuable in settings where environmental monitoring resources are limited or where timely decision-making is required to mitigate exposure risks.

Future work will focus on several key areas. First, further protein engineering could improve specificity and affinity, potentially reducing cross-reactivity with phosphate and other ligands. Second, expanded field validation across a wider range of soil types, water sources, and environmental conditions will be necessary to fully establish robustness and reliability. Third, the modular design of the device opens the possibility of multiplexed detection of additional pollutants by incorporating other binding proteins and fluorophores. Finally, long-term stability studies of both biosensor proteins and the device components will be essential for real-world deployment.

## Figures and Tables

**Table 1:** Fluorescence changes in PhnD177N∆17C conjugates in response to glyphosate.

Fluorophore	% Change	λ_max_ (nm)
**Acrylodan**	−212	460
**Coumarin**	−18	470
**AlexaFluor488**	+300	524
**TMR**	0	N/A
**Texas Red**	−3	612

## Data Availability

The original contributions presented in this study are included in the article/Supplementary Material. Further inquiries can be directed to the corresponding author.

## Supplementary Materials

The following supporting information can be downloaded at: https://www.mdpi.com/article/10.3390/bios16040225/s1.
