# Supplementary material for: A Portable Fluorometer for the Detection of Glyphosate
Source: Biosensors (Basel). 2026 Apr 20;16(4):225. doi: 10.3390/bios16040225 (PMC13115202; doi:10.3390/bios16040225)
Supplement: Supplementary file 1 [file biosensors-16-00225-s001.zip › Supplemetary material/Mini flouro 2 code.pdf]

```
#include <Wire.h>
#include <Adafruit_AS7341.h>
#include <LiquidCrystal_I2C.h>
#include <math.h>


Adafruit_AS7341 as7341;
LiquidCrystal_I2C lcd(0x27, 16, 2);   // <-- I2C LCD


// Pin setup
const int startButtonPin = 2;
const int scrollButtonPin = 6;
const int blueLEDPin = 3;
const int uvLEDPin = 4;
const int resultLEDPin = 5;


int ch = 0;
bool canSelect = false;
bool canTest = false;
bool blankCollected = false;


const char* settings[2] = {"Glyphosate", "Custom"};
const char* channels[10] = {"F1", "F2", "F3", "F4", "F5", "F6", "F7",
"F8", "CLEAR", "NIR"};


float blankAvg1, blankAvg2, sampleAvg1, sampleAvg2;
float ratio1, ratio2;
float channelData;


void setup() {
  Serial.begin(9600);
  while (!Serial);


  lcd.init();           // <-- I2C LCD init
  lcd.backlight();      // <-- Turn on backlight
  lcd.clear();
  lcd.print("Initializing...");
```

```
  if (!as7341.begin()) {
    lcd.setCursor(0, 1);
    lcd.print("Sensor Error!");
    while (1);
  }


  as7341.setATIME(1000);
  as7341.setASTEP(2000);
  as7341.setGain(AS7341_GAIN_512X);


  pinMode(blueLEDPin, OUTPUT);
  pinMode(uvLEDPin, OUTPUT);
  pinMode(resultLEDPin, OUTPUT);
  pinMode(startButtonPin, INPUT_PULLUP);
  pinMode(scrollButtonPin, INPUT_PULLUP);


  delay(1000);
  lcd.clear();
  lcd.print("Sensor ready.");
  delay(1000);
  lcd.clear();
  lcd.setCursor(0, 0);
  lcd.print("Select preset:");
  lcd.setCursor(0, 1);
  lcd.print(settings[ch]);


  canSelect = true;
  canTest = true;
}


void loop() {
  if ((digitalRead(scrollButtonPin) == LOW) && canSelect) {
    ch = (ch + 1) % 2;
    lcd.clear();
    lcd.setCursor(0, 0);
    lcd.print("Select preset:");
```

```
    lcd.setCursor(0, 1);
    lcd.print(settings[ch]);
    delay(500);
  }


  if ((digitalRead(startButtonPin) == LOW) && canTest) {
    if (settings[ch] == "Glyphosate") {
      if (!blankCollected) {
        glyphosateBlank();
      } else {
        glyphosateSample();
      }
    }
  }
}


// Timed LED Sampling Function (10s duration, 200ms interval)
float collectLEDData(uint8_t ledPin, uint8_t channel) {
  float sum = 0;
  int sampleCount = 0;
  digitalWrite(ledPin, HIGH);
  unsigned long startTime = millis();
  while (millis() - startTime < 10000) {
    as7341.readAllChannels();
    sum += as7341.getChannel(channel);
    sampleCount++;
    delay(100);
  }
  digitalWrite(ledPin, LOW);
  return sum / sampleCount;
}

void tripleBlink() {
  digitalWrite(resultLEDPin, HIGH);
  delay(150);
  digitalWrite(resultLEDPin, LOW);
  delay(150);

  digitalWrite(resultLEDPin, HIGH);
  delay(150);
  digitalWrite(resultLEDPin, LOW);
```

```
  delay(150);

  digitalWrite(resultLEDPin, HIGH);
  delay(150);
  digitalWrite(resultLEDPin, LOW);
  delay(600);  // pause before repeating
}

void doubleBlink() {
  digitalWrite(resultLEDPin, HIGH);
  delay(200);
  digitalWrite(resultLEDPin, LOW);
  delay(200);

  digitalWrite(resultLEDPin, HIGH);
  delay(200);
  digitalWrite(resultLEDPin, LOW);
  delay(600);  // pause before repeating
}

void errorBlink() {
  digitalWrite(resultLEDPin, HIGH);
  delay(150);
  digitalWrite(resultLEDPin, LOW);
  delay(150);
}

void glyphosateBlank() {
  canSelect = false;
  canTest = false;
  lcd.clear();
  lcd.setCursor(0, 0);
  lcd.print("Taking blank");
  lcd.setCursor(0, 1);
  lcd.print("at 480nm...");
  blankAvg1 = collectLEDData(uvLEDPin, AS7341_CHANNEL_480nm_F3);


  lcd.clear();
  lcd.setCursor(0, 0);
  lcd.print("Taking blank");
  lcd.setCursor(0, 1);
  lcd.print("at 515nm...");
```

```
  blankAvg2 = collectLEDData(blueLEDPin, AS7341_CHANNEL_515nm_F4);


  lcd.clear();
  lcd.print("Blanks collected");
  delay(1500);


  lcd.clear();
  lcd.print("Blank 1:");
  lcd.setCursor(0, 1);
  lcd.print(blankAvg1);
  delay(3000);


  lcd.clear();
  lcd.print("Blank 2:");
  lcd.setCursor(0, 1);
  lcd.print(blankAvg2);
  delay(3000);


  blankCollected = true;
  lcd.clear();
  lcd.setCursor(0, 0);
  lcd.print("Insert sample");
  lcd.setCursor(0, 1);
  lcd.print("& press button.");
  canTest = true;
}


void glyphosateSample() {
  canTest = false;
  lcd.clear();
  lcd.setCursor(0, 0);
  lcd.print("Taking sample");
  lcd.setCursor(0, 1);
  lcd.print("at 480nm...");
  sampleAvg1 = collectLEDData(uvLEDPin, AS7341_CHANNEL_480nm_F3);


  lcd.clear();
```

```
  lcd.setCursor(0, 0);
  lcd.print("Taking sample");
  lcd.setCursor(0, 1);
  lcd.print("at 515nm...");
  sampleAvg2 = collectLEDData(blueLEDPin, AS7341_CHANNEL_515nm_F4);


  ratio1 = sampleAvg1 / blankAvg1;
  ratio2 = sampleAvg2 / blankAvg2;


  lcd.clear();
  lcd.print("Samples collected");
  delay(1500);


  lcd.clear();
  lcd.print("Sample 1:");
  lcd.setCursor(0, 1);
  lcd.print(sampleAvg1);
  delay(3000);


  lcd.clear();
  lcd.print("Sample 2:");
  lcd.setCursor(0, 1);
  lcd.print(sampleAvg2);
  delay(3000);


  lcd.clear();
lcd.print("480nm Ratio:");
lcd.setCursor(0, 1);
lcd.print(ratio1);

// ----- LED result indicator on D5 -----
if (sampleAvg1 == 0 || blankAvg1 == 0) {
    unsigned long tStart = millis();
    while (millis() - tStart < 5000) errorBlink();
}
else if (ratio1 > 3) {
    unsigned long tStart = millis();
    while (millis() - tStart < 5000) tripleBlink();
```

```
}
else if (ratio1 >= 1.1) {
    unsigned long tStart = millis();
    while (millis() - tStart < 5000) doubleBlink();
}
else {
    digitalWrite(resultLEDPin, HIGH);
    delay(5000);
    digitalWrite(resultLEDPin, LOW);
}



  lcd.clear();
lcd.print("515nm Ratio:");
lcd.setCursor(0, 1);
lcd.print(ratio2);

// ----- LED result indicator on D5 -----
if (sampleAvg2 == 0 || blankAvg2 == 0) {
    // ERROR: cannot divide
    unsigned long tStart = millis();
    while (millis() - tStart < 5000) {
        errorBlink();
    }
}
else if (ratio2 > 3) {
    // STRONG POSITIVE: triple blink
    unsigned long tStart = millis();
    while (millis() - tStart < 5000) {
        tripleBlink();
    }
}
else if (ratio2 >= 1.1) {
    // BORDERLINE: double blink
    unsigned long tStart = millis();
    while (millis() - tStart < 5000) {
        doubleBlink();
    }
}
else {
    // NEGATIVE: solid ON
    digitalWrite(resultLEDPin, HIGH);
```

```
    delay(5000);
    digitalWrite(resultLEDPin, LOW);
}



  Serial.println(blankAvg1);
  Serial.println(blankAvg2);
  Serial.println(sampleAvg1);
  Serial.println(sampleAvg2);
  Serial.println(ratio1);
  Serial.println(ratio2);


  blankCollected = false;
  canSelect = true;
  canTest = true;
}


void getChannelData() {
  if (as7341.readAllChannels()) {
    if (channels[ch] == "F1") channelData =
as7341.getChannel(AS7341_CHANNEL_415nm_F1);
    else if (channels[ch] == "F2") channelData =
as7341.getChannel(AS7341_CHANNEL_445nm_F2);
    else if (channels[ch] == "F3") channelData =
as7341.getChannel(AS7341_CHANNEL_480nm_F3);
    else if (channels[ch] == "F4") channelData =
as7341.getChannel(AS7341_CHANNEL_515nm_F4);
    else if (channels[ch] == "F5") channelData =
as7341.getChannel(AS7341_CHANNEL_555nm_F5);
    else if (channels[ch] == "F6") channelData =
as7341.getChannel(AS7341_CHANNEL_590nm_F6);
    else if (channels[ch] == "F7") channelData =
as7341.getChannel(AS7341_CHANNEL_630nm_F7);
    else if (channels[ch] == "F8") channelData =
as7341.getChannel(AS7341_CHANNEL_680nm_F8);
    else if (channels[ch] == "CLEAR") channelData =
as7341.getChannel(AS7341_CHANNEL_CLEAR);
    else channelData = as7341.getChannel(AS7341_CHANNEL_NIR);
  }
}
```
