# Supplementary material for: A Portable Fluorometer for the Detection of Glyphosate
Source: Biosensors (Basel). 2026 Apr 20;16(4):225. doi: 10.3390/bios16040225 (PMC13115202; doi:10.3390/bios16040225)
Supplement: Supplementary file 1 [file biosensors-16-00225-s001.zip › Supplemetary material/code_program for mini fluoro gen 1.pdf]

Program for operating the device

```
#include <Wire.h>
#include <Adafruit_Sensor.h>
#include <Adafruit_TSL2591.h>
Adafruit_TSL2591 tsl = Adafruit_TSL2591(2591);
const int buttonPin = 2; // Pin for the push button
const int redLEDPin = 3; // Pin for the red LED
const int greenLEDPin = 4; // Pin for the green LED
const int blueLEDPin = 5; // Pin for the blue LED
bool firstPress = true;
bool collecting = false;
unsigned long startTime;
const int collectTime = 10000; // Collect data for 10 seconds
float minThreshold = 1.1;
float maxThreshold = 3;
float blank = 0;
float sample = 0;
float prevVisible;
void configureSensor() {
 tsl.setGain(TSL2591_GAIN_MAX);
 tsl.setTiming(TSL2591_INTEGRATIONTIME_100MS);
}
void setup() {
 Serial.begin(9600);
 pinMode(buttonPin, INPUT);
 pinMode(redLEDPin, OUTPUT);
 pinMode(greenLEDPin, OUTPUT);
 pinMode(blueLEDPin, OUTPUT);
 Serial.println("Finding sensor...");

 if (tsl.begin()) {
 Serial.println("Found a TSL2591 sensor");
 } else {
 Serial.println("No sensor found ... check your wiring?");
 while (1);
 }
 Serial.println("Configuring sensor...");
 configureSensor();
 Serial.println("Sensor configured");
 Serial.println("Insert blank, then press the button to continue.");
}
float calculateStandardDeviation(float data[], int n, float mean) {
 float sum = 0;
 for (int i = 0; i < n; i++) {
 sum += pow(data[i] - mean, 2);
 }
 return sqrt(sum / n);
}
```

```
void loop() {
 if (digitalRead(buttonPin) == HIGH && !collecting) {
 collecting = true;
 startTime = millis();
 float totalVisible = 0;
 int readings = 0;
 float visibleValues[100]; // Assuming a maximum of 100 readings within 10 seconds
 delay(100);
 digitalWrite(greenLEDPin, LOW);
 digitalWrite(redLEDPin, LOW);
 digitalWrite(redLEDPin, HIGH);
 delay(100);
 digitalWrite(redLEDPin, LOW);
 Serial.println("Button pressed. Collecting data for 10 seconds.");
 digitalWrite(blueLEDPin, HIGH);

 while (millis() - startTime < collectTime) {
 prevVisible = totalVisible;
 uint16_t x = tsl.getLuminosity(TSL2591_VISIBLE);
 totalVisible += x;
 visibleValues[readings] = x;
 readings++;
 delay(100); // Read every 100ms
 Serial.print(totalVisible - prevVisible);
 Serial.print(", ");
 }
 digitalWrite(greenLEDPin, HIGH);
 delay(100);
 digitalWrite(greenLEDPin, LOW);
 delay(50);
 Serial.println("");
 digitalWrite(blueLEDPin, LOW);

 float averageVisible = totalVisible / readings;
 float stdDev = calculateStandardDeviation(visibleValues, readings, averageVisible);

 if (firstPress) {
 blank = averageVisible;
 Serial.println("Blank value collected.");
 Serial.print("Average Blank value: ");
 Serial.println(blank);
 Serial.print("Blank standard deviation: ");
 Serial.println(stdDev);
 firstPress = false;
 Serial.println("Insert sample, then press the button to continue.");
 } else {
 sample = averageVisible;
 Serial.println("Sample value collected.");
```

```
Serial.print("Average Sample value: ");
Serial.println(sample);
Serial.print("Sample standard deviation: ");
Serial.println(stdDev);

if (sample != 0) {
float ratio = sample / blank;
Serial.print("Ratio (sample/blank): ");
Serial.println(ratio);

// Control LEDs based on the ratio
if (ratio > maxThreshold) {
digitalWrite(redLEDPin, HIGH);
digitalWrite(greenLEDPin, HIGH);
Serial.println("Both LEDs ON");
} else if (ratio >= minThreshold) {
digitalWrite(redLEDPin, HIGH);
digitalWrite(greenLEDPin, LOW);
Serial.println("Red LED ON");
} else {
digitalWrite(redLEDPin, LOW);
digitalWrite(greenLEDPin, HIGH);
Serial.println("Green LED ON");
}
} else {
Serial.println("Sample value is 0, cannot divide by zero");
}

Serial.println("Insert blank and press the button to test again.");
// Reset for next cycle
firstPress = true;
}

collecting = false;
delay(1000); // Debounce delay
}

}
```
