# Supplementary material for: A Portable Fluorometer for the Detection of Glyphosate
Source: Biosensors (Basel). 2026 Apr 20;16(4):225. doi: 10.3390/bios16040225 (PMC13115202; doi:10.3390/bios16040225)
Supplement: Supplementary file 1 [file biosensors-16-00225-s001.zip › Supplemetary material/Blue LED emission.docx]

Supplemental material

Supp. Fig. 1.


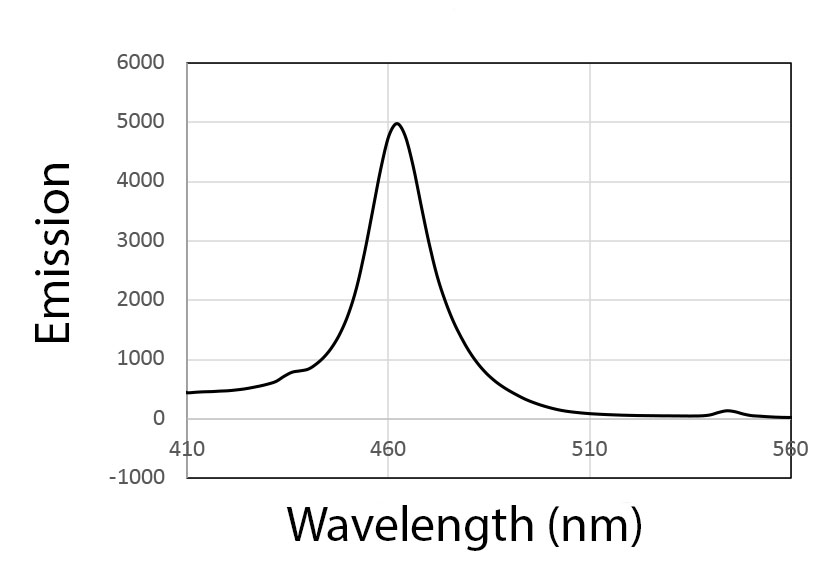


**Supplemental Fig. 1:** Peak emission of the blue LED (462nm) used as the excitation source for the device
