# Supplementary figures and images for: A Portable Fluorometer for the Detection of Glyphosate
Source: Biosensors (Basel). 2026 Apr 20;16(4):225. doi: 10.3390/bios16040225 (PMC13115202; doi:10.3390/bios16040225)

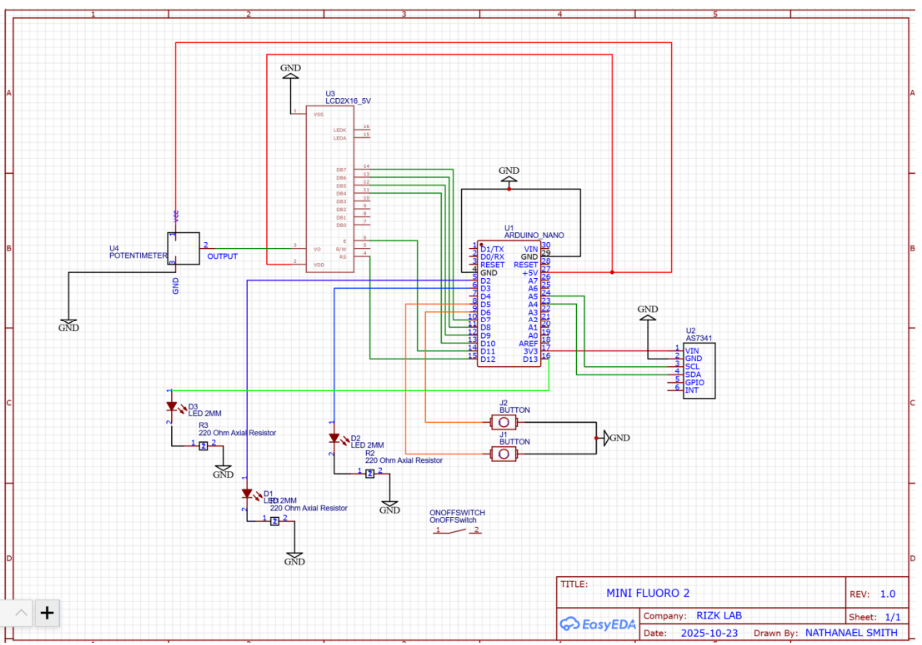

Supplement: Supplementary file 1 [file biosensors-16-00225-s001.zip › Supplemetary material/Wiring diagram 2.0.png]

2.0 Mini Flourometer device : (Old Wire Set Up)

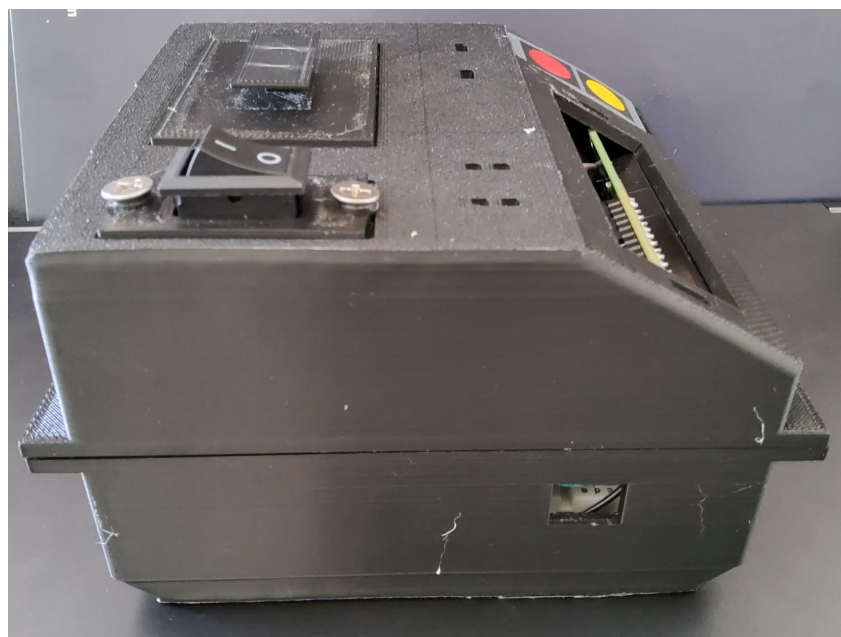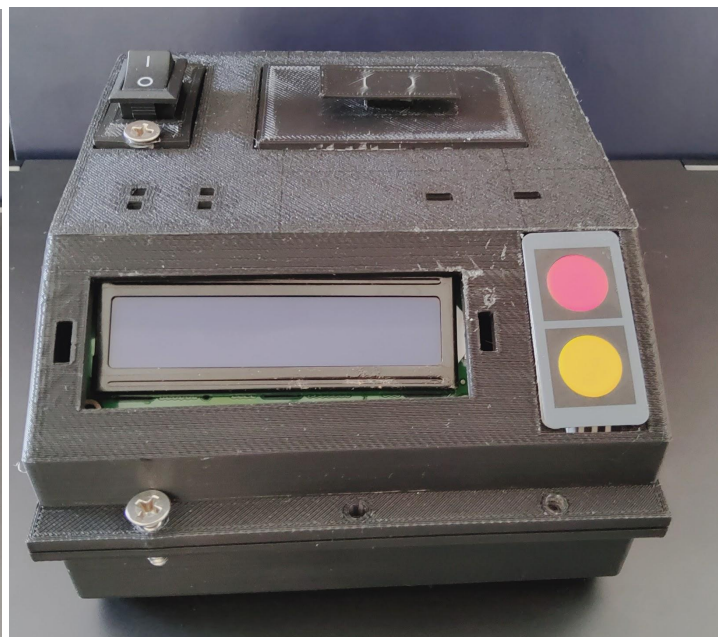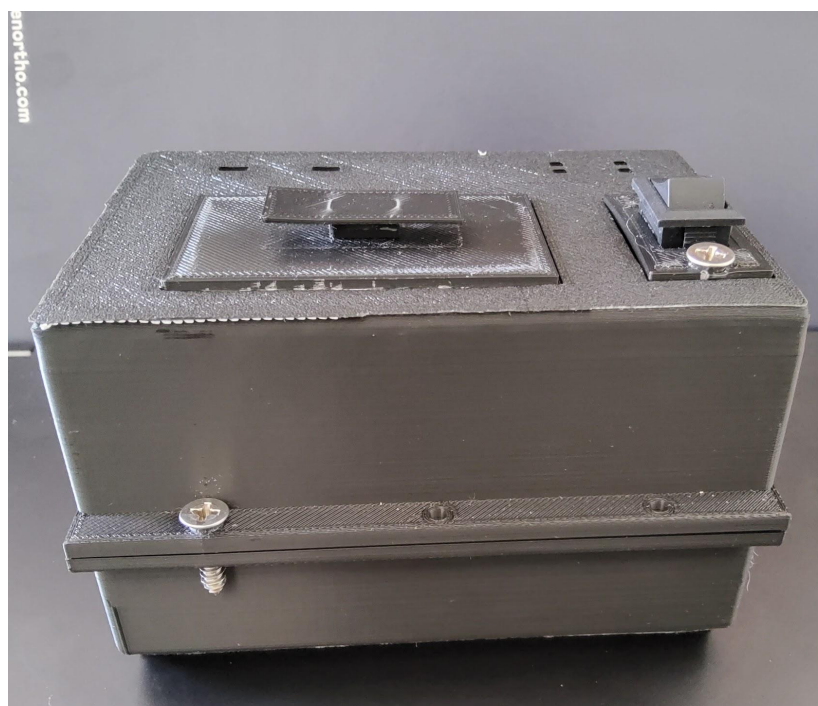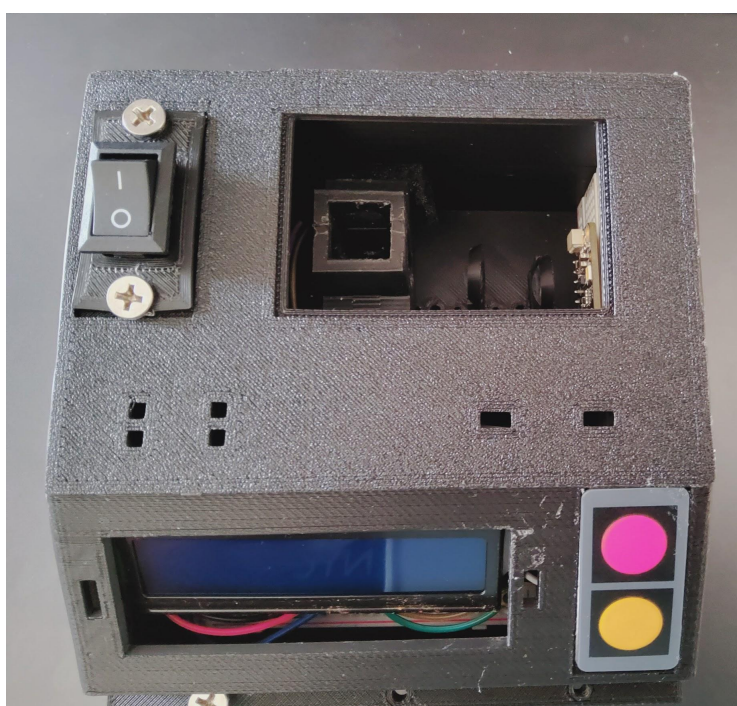

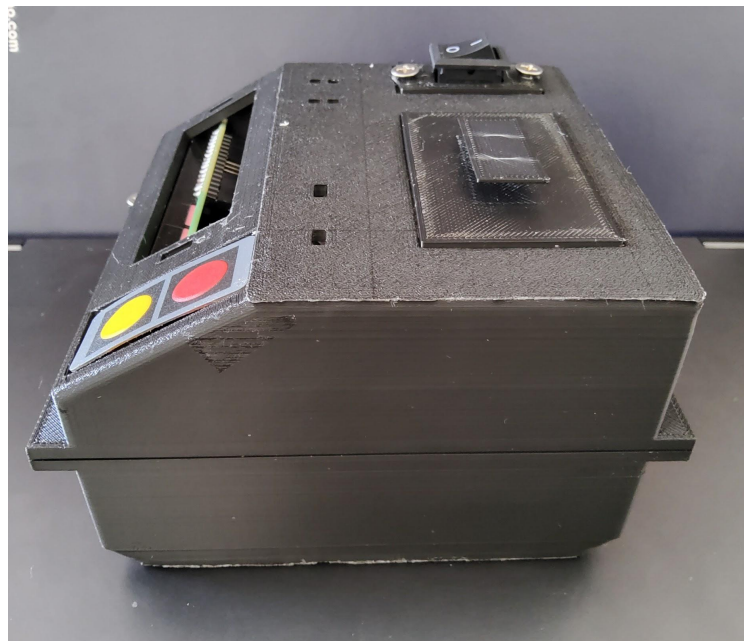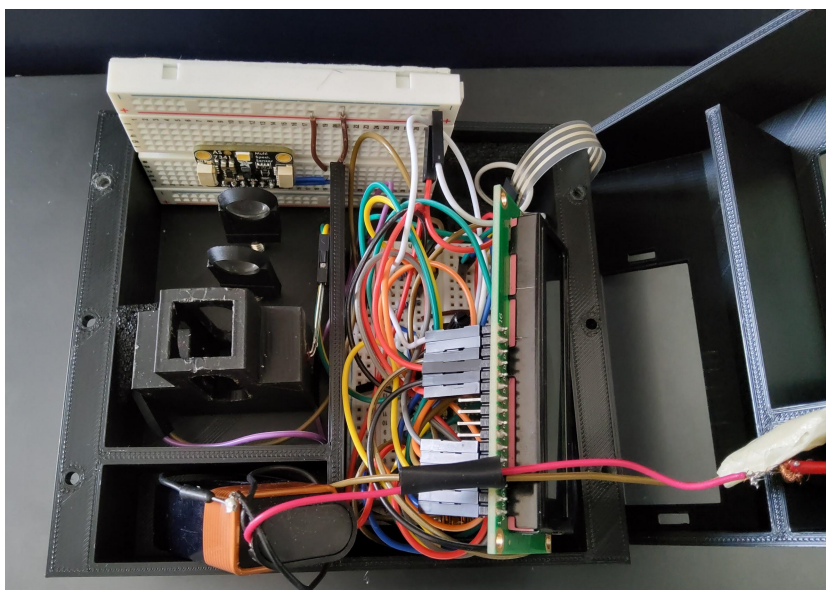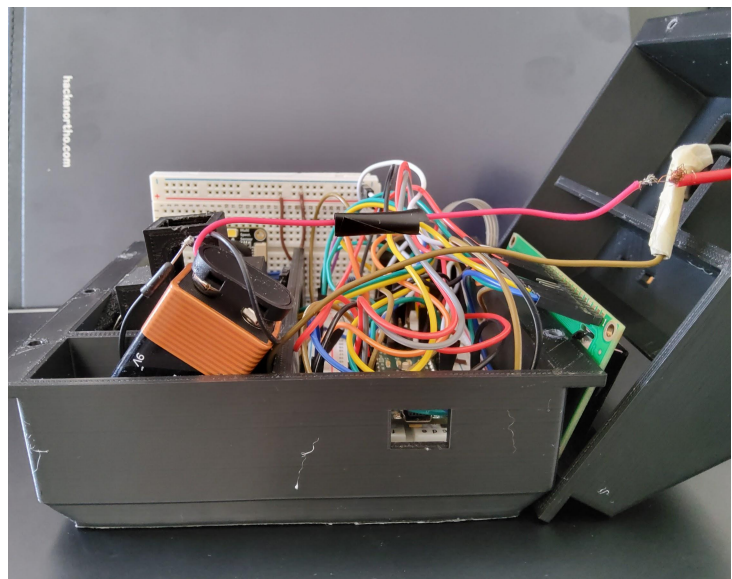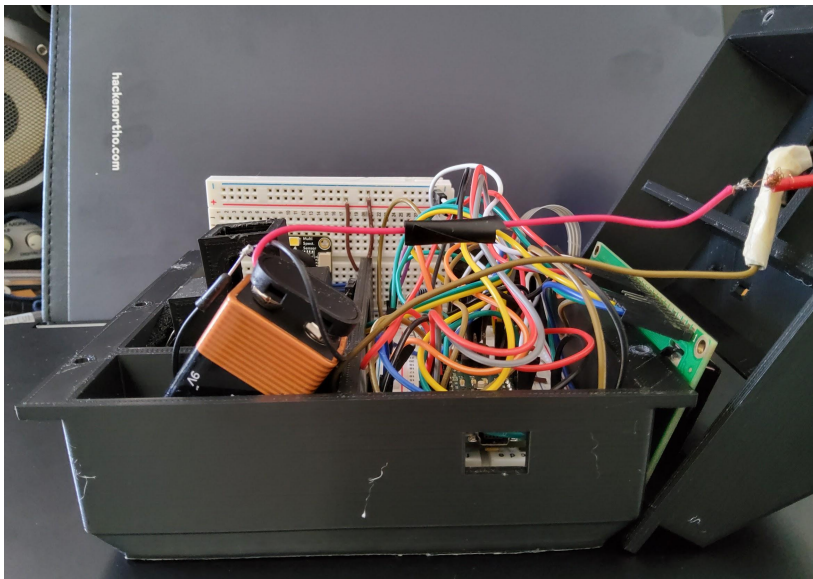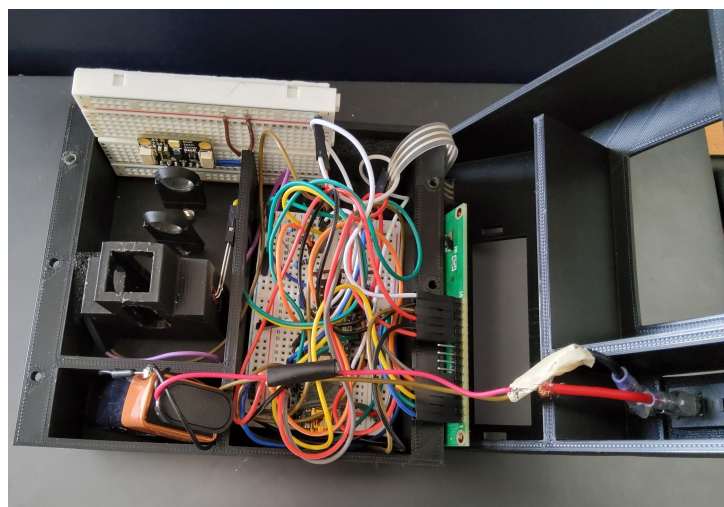

Supplement: Supplementary file 1 [file biosensors-16-00225-s001.zip › Supplemetary material/Flourometer Pictures.pdf]

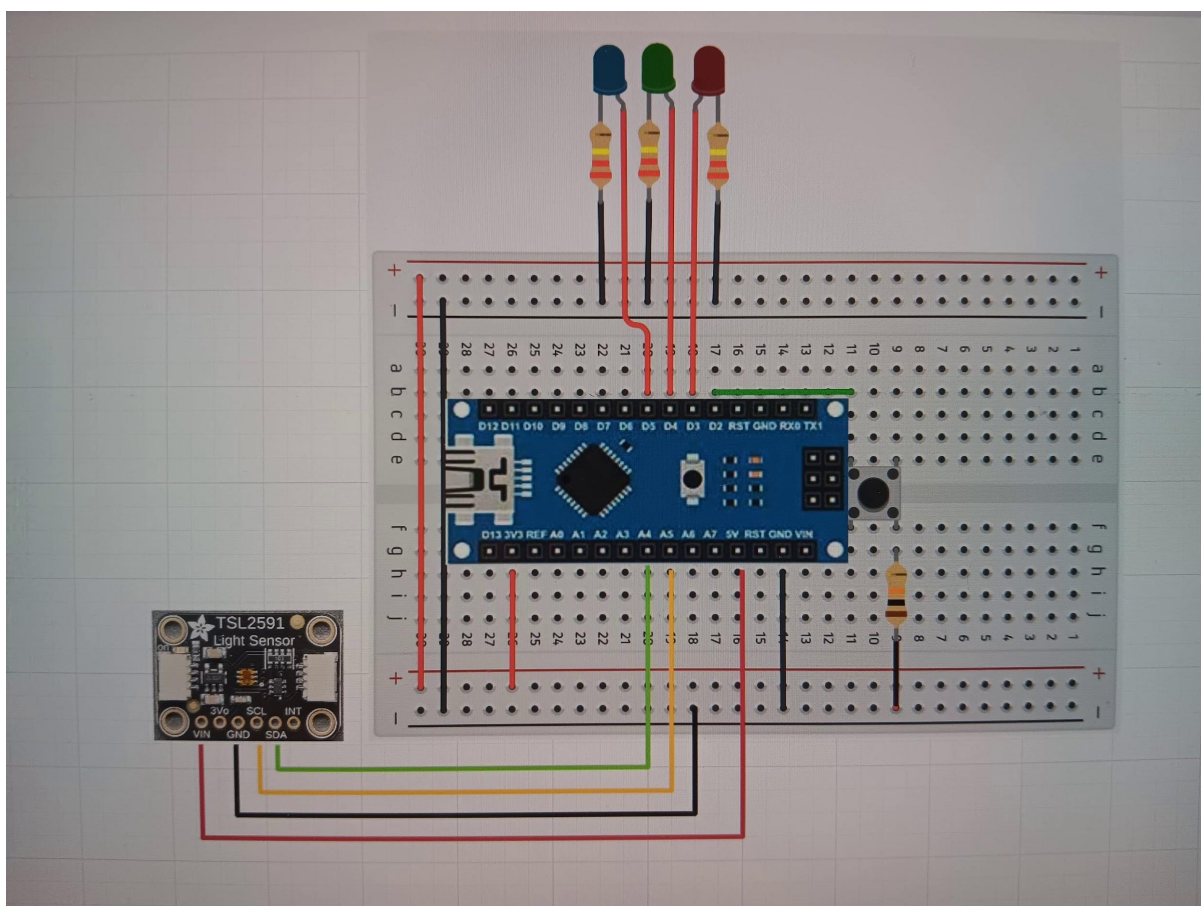

Supplement: Supplementary file 1 [file biosensors-16-00225-s001.zip › Supplemetary material/wiring diagram mini fluoro 1.pdf]
